# From Bench to Bedside: Ethical and Clinical Best Practices for Genome Editing Applications

**DOI:** 10.3390/ijms27031484

**Published:** 2026-02-02

**Authors:** María Ortiz-Bueno, Federica Zinghirino, Pilar Puig Serra, Kyriaki Paschoudi, Lluis Montoliu, Erden Atilla, Yonglun Luo, Alessia Cavazza, Carsten W. Lederer, Karim Benabdellah

**Affiliations:** 1 GENYO, Centre for Genomics and Oncological Research: Pfizer, University of Granada, 18016 Granada, Spain; 2 Department of Biochemical Engineering, University College London (UCL), London WC1E 6BT, UK; f.zinghirino@ucl.ac.uk; 3 Molecular Cytogenetics and Genome Editing Unit, Human Cancer Genetics Program, Spanish National Cancer Research Center (CNIO), 28029 Madrid, Spain; 4 Department of Genetics, Development and Molecular Biology, School of Biology, Aristotle University of Thessaloniki, 54124 Thessaloniki, Greece; paschoudik@gmail.com; 5 Gene and Cell Therapy Center, Hematology Clinic, George Papanikolaou Hospital, 57010 Thessaloniki, Greece; 6 Department of Molecular and Cellular Biology, National Centre for Biotechnology (CNB-CSIC), 28049 Madrid, Spain; 7 Rare Diseases Networking Biomedical Research Centre (CIBERER-ISCIII), 28029 Madrid, Spain; 8 Transplantation and Cellular Therapy, Department of Medicine, University of Miami Miller School of Medicine, Miami, FL 33136, USA; 9 Department of Biomedicine, Aarhus University, 8000 Aarhus, Denmark; 10 Steno Diabetes Center Aarhus, Aarhus University Hospital, 8200 Aarhus, Denmark; 11 Molecular and Cellular Immunology Section, Department of Infection, Immunity & Inflammation, Great Ormond Street Institute of Child Health, University College London, London WC1N 1DZ, UK; a.cavazza@ucl.ac.uk; 12 Department of Medical and Surgical Sciences for Children and Adults, School of Medicine, University of Modena and Reggio Emilia, 41124 Modena, Italy; 13 Department of Blood Disorder Genetics & Thalassemia, The Cyprus Institute of Neurology & Genetics, Nicosia 2371, Cyprus

**Keywords:** genome editing, CRISPR, best practices, clinical applications, off-target effects

## Abstract

Genome editing (GE) has transformed medicine by allowing precise changes to DNA, offering potential treatments for a range of inherited and acquired disorders. Several technologies support these advances, including zinc-finger nucleases (ZFNs), transcription activator-like effector nucleases (TALENs), and clustered regularly interspaced short palindromic repeats (CRISPR)-based systems, of which the latter has emerged as the most accessible, versatile, and popular. While GE holds great promise, its clinical use requires careful attention to safety, ethics and regulatory standards. Inadvertent on- and off-target DNA alterations and unintended modification of non-target cells pose major technical challenges, while bioethical considerations and the need for harmonized safety standards create regulatory challenges. The Food and Drug Administration (FDA) and European Medicines Agency (EMA), as regulatory agencies for key advanced therapy markets, provide detailed guidance on these aspects, emphasizing rigorous preclinical testing, patient monitoring, ethical consent, and compliance with legal frameworks. This concise review summarizes what is currently published in the scientific literature and recommended by regulatory agencies, providing an overview of the responsible clinical application of GE, with emphasis on patient safety, adherence to regulatory guidance, and ethical practice.

## 1. Introduction

Genome editing (GE) stands as one of the most groundbreaking advances in molecular biology, enabling the precise manipulation of DNA to correct, eliminate or introduce genetic material. The field accelerated markedly with the adoption and modular extension of clustered regularly interspaced short palindromic repeats/CRISPR-associated proteins (CRISPR/Cas) systems, which function as RNA-guided ribonucleoprotein particles (RNPs) combining simplicity, efficiency, and accuracy for research and clinical applications. Recent clinical successes and current academic research as well as commercial product pipelines indicate that its contributions to modern medicine will be immense. Existing clinical landmarks include treatments for genetic disorders, such as sickle cell anemia and β-thalassemia [1], improving cancer immunotherapy, fostering advances in regenerative medicine and, recently, fast-tracking personalized advanced therapy for severe carbamoyl-phosphate synthetase 1 (CPS1) deficiency [2]. Unlike any other technology, GE facilitates personalized medical approaches and aligns treatments with individual genetic profiles to achieve greater therapeutic outcomes [3]. However, the application of GE demands careful adherence to best practices to address inherent challenges. For in vivo applications, off-target cell modifications remain a risk, as with other advanced therapy platforms delivered in vivo. Specific for GE, off-target effects, where unintended regions of the genome are altered, and unintended on-target alterations and recombination events remain a critical safety concern [4,5]. Yet, emerging technologies, such as base editing and prime editing, offer safer alternatives by enabling precise DNA changes independent of double-strand breaks (DSB), thereby inherently reducing unwanted genetic damage [6,7] and allowing ongoing safety optimization [8].

Additionally, ethical considerations are essential for responsible use of GE, which calls for international collaboration to establish and harmonize guidelines that promote equity, accessibility and, thus, societal acceptance. Regulatory frameworks must prioritize patient safety, transparency, and public trust while ensuring that benefits of these transformative technologies are distributed fairly. To address these issues, the COST Action GenE-HumDi has been created [9], facilitating discussions on ethical, regulatory, and societal aspects through meetings with regulators and bilateral exchanges. Only by addressing these facets can the potential of GE be realized to reshape medicine, improve global health, and reduce disparities [10].

In summary, the objective of this concise review is to provide a clear, evidence-based overview of best practices for the clinical application of GE, presenting the main narrative in non-technical language. Its focus is on promoting the safe, effective, and ethically responsible use of GE in therapeutic settings. To allow that focus while keeping the main text accessible to readers at different levels of proficiency in molecular sciences, this article employs info boxes to give additional molecular insights. The document emphasizes strategies to ensure patient safety, including CRISPR-specific safety checks, minimizing off-target effects, and optimizing delivery systems. Ethical considerations, patient selection, and informed consent processes are also addressed, alongside key regulatory frameworks such as those of the FDA (Food and Drug Administration, the main agency in the United States responsible for approving medicines and ensuring their safety) and the EMA (European Medicines Agency, the European Union agency that evaluates and supervises medicines for human use). Overall, adherence to these best practices is essential for the successful and responsible implementation of GE in clinical practice.

## 2. GE Achievements and Potential

GE has emerged as a powerful precision tool to correct the genetic changes that cause diseases. For a summary of key terms relevant to this section, see Box 1. Conditions caused by a single defective gene—such as sickle cell anemia, β-thalassemia, cystic fibrosis, Duchenne muscular dystrophy, and hemophilia—can now be treated at the DNA level using advanced technologies like CRISPR/Cas, base editors, and prime editors. In recent clinical trials, doctors have been able to take a patient’s own stem cells, edit them outside the body and return them to the patient, restoring normal function [11]. These approaches have shown great promise while keeping unintended changes to a minimum. To date, GE approaches have seen their most effective and clinically advanced application in non-malignant rare diseases conventionally treatable by hematopoietic stem and progenitor cell (HSPC) transplantation, such as blood disorders and many metabolic and neuronal disorders [12]. Amid a rapidly expanding body of studies feeding into industrial pipelines, these strategies enable correction of genetic defects underlying diseases such as hemoglobinopathies, be it by precision repair of causative mutations, by activating therapeutic disease modifiers, or by precise gene addition for functional correction [13].

Additionally, GE is making a difference in cancer treatment. It allows scientists to precisely modify immune cells, such as T-cells, to recognize and attack cancer cells more easily. In some approaches, GE can even target tumor cells directly. These strategies are being explored for both blood cancers and solid tumors, opening new possibilities for more effective and personalized therapies [11].

Engineered immune cells are powerful anticancer tools, but their development required overcoming challenges related to specificity, immune cross-reactivity, and limited cellular persistence. Use of the patient’s own immune cells resolves some of the immunological complications but comes at a high cost for treatment, while for solid tumors, access to engineered immune cells represents an additional impediment. However, across the board, application of GE is showing potential to allow significant progress toward more affordable, safer and more effective engineered immunotherapies. In this context, GE is being applied to T cells and natural killer (NK) cells to develop advanced immunotherapies for blood cancers. These engineered immune cells are better able to recognize and destroy diseased or malignant cells, for safer precision engineering of specificity, while allowing enhancement of immune function [14,15,16]. More importantly and unique to GE-engineered immune cells, the ability to introduce multiple simultaneous changes allows reprogramming of immune cell specificity with enhancement of immune function and the ability to create off-the-shelf immune cells that are applicable to different patients. The latter will predictably allow scalable production for lower cost and provide engineered immune cells for critically ill patients without delay [17,18].

GE also allows scientists to create accurate models of cancer, identify new treatment targets, and edit genes directly that drive tumor growth. The latter would allow direct application of GE to malignancies as potential supportive therapy, e.g., by inactivating *KRAS*, a gene that produces a protein called K-Ras, which acts as a molecular switch controlling cell growth and division [19]. In this sense, CRISPR can be used to “switch off” or activate a range of genes involved in tumor development, including genes that control cell division, protect against cancer or regulate tumor growth pathways. By adjusting these key genes, researchers aim to slow tumor growth, restore normal cell function, and allow more effective treatments [20]. Similarly, in other solid tumors such as lung cancer, CRISPR/Cas has been applied to address therapy resistance associated with key oncogenic drivers [20,21].

Box 1 **Key terms of GE and vector technology.**

**AAV (Adeno-Associated Virus):** Vectors with strong tissue tropism and non-integrative profiles, widely used in vivo despite limited cargo capacity.
**AdV (Adenoviral Vectors):** Vectors with large payload capacity and transient expression, suited for ex vivo and HSPC-targeted in vivo applications despite potential immunogenicity.
**Base editor:** Fusion of Cas nickase and deaminase, enabling single-nucleotide transitions independent of double-strand breaks.
**Cas endonuclease:** Fully active Cas enzyme (e.g., Cas9, Cas12a) capable of introducing double-strand breaks for classical gene disruption or template-based precision repair at targets determined by a guide RNA.
**Cas nickase:** Modified Cas9 with one active nuclease domain—typically the D10A mutation in the RuvC domain (nCas9-D10A) or H840A mutation in the HNH domain (nCas9-H840A)—inducing single-strand nicks to reduce DSB-related genotoxicity and potentially enhance base or prime editing.
**High-fidelity Cas9 variants:** Engineered Cas9 nucleases that improve specificity, though usually with some reduction in *on-target* activity, e.g., by alanine substitutions to replace positively charged residues and reduce unspecific DNA interactions (R780, K848, K855, H982 → eSpCas9) or by aspartic-acid substitutions to disrupt a mismatch stabilizing RuvC-domain loop (Y515, N517, L518, Q519, R520, F522, Y523 → SuperFi-Cas9).
**CRISPR/Cas:** RNA-guided nuclease system adapted from bacterial immunity, enabling speedily designed, programmable, and scalable GE.
**DSB (DNA double-strand break):** Introduced by first-generation genome editors as trigger for genome modification.
**Electroporation/Nucleofection:** Electrical permeabilization methods enabling intracellular introduction of RNPs or nucleic acids, standard in ex vivo protocols.
**EV (Extracellular Vesicle):** Naturally secreted vesicle investigated for low-immunogenicity delivery of GE cargos.
**Ex vivo editing:** Isolation, genetic modification, and reinfusion of patient-derived cells, commonly used in cell-based therapies.
**gRNA (guide RNA):** DNA-binding component of CRISPR-based editors for target recognition by Watson–Crick base pairing.
**HDR (Homology Directed Repair):** Precise DSB repair mechanism that uses a homologous DNA sequence as a template to accurately repair or insert genetic material.
**IDLV (Integrase-Defective Lentiviral Vectors):** Vectors used for transient delivery, minimizing genomic integration risks.
**In vivo editing:** Direct delivery of editing systems into the patient, typically via viral vectors or lipid nanoparticles.
**LNP (Lipid Nanoparticle):** Nanosized particle composed mainly of lipids that encapsulate mRNA or RNPs, validated for systemic CRISPR delivery.
**NHEJ (Non-Homologous End Joining):** Predominant error-prone DSB repair pathway resulting in insertions/deletions (indels) or chromosomal rearrangement, used for targeted gene disruption.
***Off-target:*** Locus of unintended genomic modifications based on sequence similarity to the intended target sequence; a major safety concern in clinical editing.
***On-target:*** Genomic locus of intended modification, based on specificity conferred by the DNA-binding component of the genome editor.
**PAM (Protospacer Adjacent Motif):** Short DNA motif required for Cas enzyme recognition and cleavage (e.g., 5′-NGG-3′ for SpCas9), defining permissible editing sites.
**Prime editor:** Cas nickase fused to reverse transcriptase and guided by pegRNA, enabling insertions, deletions, or substitutions independent of donor DNA or DSBs.
**TALEN (Transcription Activator-Like Effector Nucleases):** Dimeric nucleases that recognize single nucleotides via modular repeats, offering targeting flexibility with a clear amino-acid code for DNA binding, based on cloning-based re-targeting.
**ZFN (Zinc Finger Nucleases):** Dimeric engineered nucleases guided by zinc-finger DNA-binding domains, based on empirical design and cloning-based re-targeting.

## 3. Efficiency and Safety Concerns

One of the major challenges in translating GE to the clinic is the safe and efficient delivery of editing tools to the intended target cells. Safety is the highest priority for advanced therapies, particularly considering severe adverse events observed in earlier clinical trials [22,23], which meant tragic consequences for trial subjects and in turn severe setbacks for the field itself. Complementary to and in support of the work by official regulatory bodies, safety is therefore also considered in self-governance of stakeholders, as exemplified by the Association for Responsible Research and Innovation in Genome Editing (ARRIGE) [24]. For therapeutic use of GE, safety aspects include many considerations that altogether increase predictability and avoid unwanted effects of the editing procedure. Most critically, these include efficient and precise delivery systems for GE, consideration of immune responses, trends toward in vivo delivery of GE systems, confirming safety and efficiency assessment for basic editor designs, and engineering advanced editing systems.

### 3.1. Delivery Systems for GE

**Figure 1:**
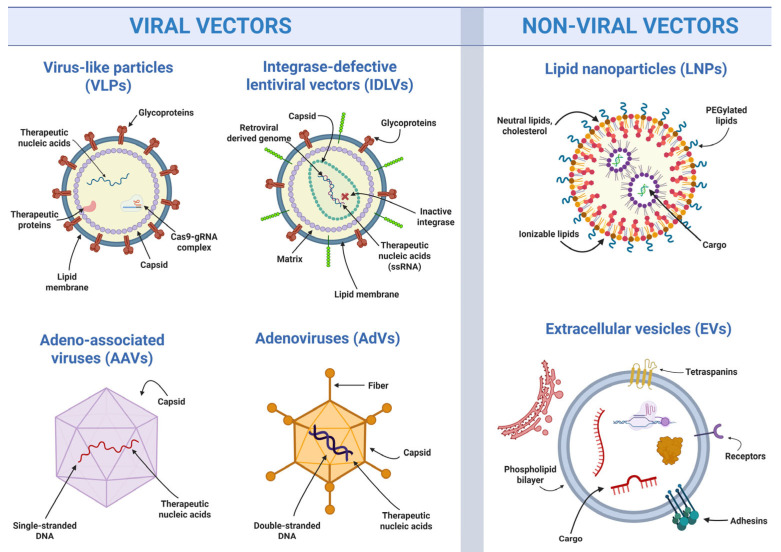
Comparison of methods for delivering genetic and therapeutic material: viral vs. non-viral vectors. The schematic provides an overview of major delivery systems used for GE, excluding mechanical transfection of naked GE components. Viral systems include virus-like particles (VLPs), integrase-defective lentiviral vectors (IDLVs), adeno-associated viral vectors (AAVs), and adenoviral vectors (AdVs). Non-viral systems include lipid nanoparticles (LNPs) and extracellular vesicles (EVs). These delivery methods differ in mechanism, cargo capacity, efficiency, and safety profile [14]. Created in BioRender. Benabdellah, K. (2026) https://BioRender.com/occx0fe (accessed on 20 January 2026) and adapted from Cavazza et al. [14].

For both efficiency and safety, GE tools should be restricted to target cells and remain active only briefly to minimize unintended editing. On this point it is important to highlight that the GenE-HumDi network has recently published a review article that summarizes the main ways scientists do this, whether editing cells outside the body (ex vivo) or directly in tissues and organs (in vivo) [14]. The underlying tools can be separated into two main categories: viral systems and non-viral systems [14], as illustrated in Figure 1. For a summary of key terms relevant to this section, see Box 1.

On the one hand, viral systems use modified viruses to carry GE tools into cells [14]. These include integrase-defective lentiviral vectors (**IDLVs**), which do not permanently integrate into DNA and suitable for dividing and non-dividing cells; adeno-associated virus vectors (**AAVs**), which are small viral vectors efficient for in vivo use but limited in cargo capacity; adenoviral vectors (**AdVs**), which can accommodate larger genetic payloads and act quickly, show natural in vivo selectivity for HSPCs [25,26] but may trigger stronger immune responses and by carrying viral double-stranded DNA pose a heightened safety concern; and virus-like particles (**VLPs**), that deliver proteins directly without carrying viral genetic material, thereby reducing risk.

On the other hand, non-viral systems avoid viral components altogether and are generally safer, though sometimes less efficient. Major examples include lipid nanoparticles (**LNPs**), tiny lipid-based bubbles that encapsulate genetic material and deliver it to cells and that have been widely used in recent mRNA therapies; extracellular vesicles (**EVs**), naturally cell-derived vesicles that can be loaded with editing tools and are well tolerated by the body; and finally, **naked GE components**, protein, RNP or RNA mixtures that are electroporated into target cells and currently represent the most effective and clinically relevant ex vivo delivery method for HSPCs [14]. Researchers in the GenE-HumDi network are continuously testing these delivery methods, sharing results, and holding meetings to compare approaches and discuss the best strategies. This ongoing work helps move GE closer to safe and effective treatments for patients.

### 3.2. Consideration of Immune Responses

Most CRISPR/Cas9 systems, including the recently FDA-approved GE therapies, rely on Cas9 nucleases derived from common human bacterial pathogens, such as *Staphylococcus aureus* and *Streptococcus pyogenes*. Multiple studies investigating pre-existing immunity to Cas9 report pre-existing humoral and T-cell immunity against Cas9 and thus the risk that Cas9-expressing cells could be targeted during GE treatments [27,28]. While anti-Cas antibodies and thus the risk of harmful inflammation are low in ocular fluid [29], Cas9-reactive antibodies, CD4^+^ and CD8^+^ T cells, and SpCas9 and SaCas9-specific cytokine production are common in the serum of healthy individuals [30]. However, reported prevalence estimates may not fully reflect the clinical relevance of Cas9-specific humoral immunity [31]. Casgevy™ application has proven to be safe to date [32], and pre-existing antibodies in rhesus macaques did not prompt an immune response to ex vivo-edited cells in either the short- or long-term [33]. Collectively, these findings indicate that at least transient ex vivo application of Cas9 protein is safe in practice. Still, there is clear indication that pre-existing adaptive immunity to Cas9 may reduce the safety and efficacy of CRISPR/Cas9 therapies in certain scenarios, which has promoted several mitigation strategies to reduce immunogenicity and is of particular importance for the nascent field of in vivo delivery of GE components by application to the bloodstream.

### 3.3. In Vivo Delivery of GE Systems

Recent years have seen the burgeoning of in vivo delivery for GE components (e.g., CRISPR nucleases plus guides delivered by AAVs, AdVs, LNPs, VLPs, etc.) [26,34,35,36], which may fundamentally simplify development and deployment of GE-based therapies compared with ex vivo editing [37]. In contrast to bespoke patient-specific cell products, in vivo approaches enable off-the-shelf vector formulations with standardized QC and batch release, like conventional biologics. This avoids collection, processing, and shipping of target cells to centralized facilities and back again, reducing chain-of-identity complexity and enabling more decentralized application at qualified treatment centers. These logistics advantages reduce cost but are especially important where timely access is critical or where cell-handling infrastructure is limited. Tolerability can also improve for indications currently relying on ex vivo edited hematopoietic stem/progenitor cells (HSPCs), because in vivo approaches may avoid (or reduce) the need for myeloablative conditioning, a major contributor to morbidity, treatment-related mortality, infertility, and infectious complications [38].

At the same time, in vivo editing introduces new risks, including unintended editing of non-target tissues, such as inadvertent exposure of gonadal tissue with theoretical germline implications [39], reduced efficiency due to hepatic sequestration of GE delivery complexes (particularly for LNPs) [40], and diminished efficiency or inflammatory responses resulting from prolonged exposure and immunogenicity of GE or delivery elements, such as AAV and AdV, in the bloodstream [41]. Several synergistic strategies are being pursued to address all these concerns, with significant recent successes, including application of transient GE formulations [14], design of Cas proteins with reduced immunogenicity [41,42,43], immune-evasive vector designs and carrier formulations [44,45], and improved targeting of GE complexes [46]. These technical advancements of GE components are paralleled by strict regulatory oversight (see Section 4) and technical advancements also of corresponding assessment methodology.

### 3.4. Safety and Efficiency Assessment for Basic Editor Designs

For ex vivo and in vivo application of GE, design and application must be tested for minimal unintended side effects to ensure patient safety. Thus, comprehensive validations of CRISPR/Cas constructs and editing outcomes must be made. Initially, at the start of a GE experiment, scientists should consider fixed properties of the Cas protein component and carefully design the readily modifiable guide RNA (gRNA) [47]. Depending on the type and species origin, different Cas enzymes require different PAM sequences—short DNA “addresses”—which limit the freedom of choosing genome sites for editing, while preventing unintended edits at sites missing a suitable PAM sequence and thus contributing to the accuracy of GE [48]. The gRNA is a short piece of RNA that acts like a keyword search string for the CRISPR system, directing it to the exact text match in the DNA sequence that needs to be edited. By matching the gRNA to the target gene, CRISPR can cut DNA at the precise location, allowing researchers to add, remove or correct specific genetic sequences. Using advanced tools and databases to design gRNAs helps ensure they target the correct gene and avoid unintended, so-called off-target, changes elsewhere within the genome. Before editing the genome, it is therefore important to consider both PAM and gRNA properties and predict where CRISPR might accidentally cut the DNA, which could cause unintended changes. This step is called in silico off-target prediction. Scientists use specialized computer tools, such as COSMID, CRISPOR, and Cas-OFFinder, to scan the genome and identify potential off-target sites. By checking these sites in advance, researchers can reduce the risk of harmful mistakes and make GE safer and more precise [49,50]. Additionally, the utilization of techniques such as GUIDE-seq or CIRCLE-seq allows identification and mitigation of off-target effects [51,52]. Recently, development of CAST-seq has enabled identification and quantification of complex events that were missed by many other methods, including the inadvertent fusion of off-target and on-target sites [53]. A representative overview of these tools and methodologies is provided in Table 1, illustrating the diversity of current approaches, while more comprehensive analyses can be found in recent dedicated reviews [4,49,50,51,52,54].

Many existing methods are limited by site-specific amplification and fail to detect large deletions or complex rearrangements. Here, assays for the assessment of genome integrity are employed and will increasingly become part of routine regulatory requirements for safety assessments. Besides some of the above methods for double-strand break detection, these methods include CAST-seq and similar methodology for the detection of large on-target aberrations and recombination events between on- and off-target loci [4] and increasingly embrace targeted long-read sequencing for the detection of structural variants, including large deletions, insertions and complex rearrangements [58]. These methods are also complemented by routine cytogenetics detection [59] and, increasingly, by whole-genome sequencing using long- or short-read massively parallel sequencing [60,61], although their sensitivity and broader clinical implementation remain limited by read depth and cost, respectively.

Beyond these intracellular events, GE application by in vivo delivery adds the challenge of off-target cell delivery. The need to detect events in individual tissue and cell types therefore calls for in situ detection or for the use of highly sensitive detection technologies capable of identifying highly diluted editing events. Different technologies have been proposed and applied, based on tissue-specific isolated cell populations and for enrichment before whole-genome sequencing [62,63]. The need for further technological development and the corresponding challenges both have been acknowledged by the FDA and in a recent workshop of the EMA Committee for Advanced Therapies [64,65].

Beyond basic retargeting and assessment of existing GE and delivery components, followed by selection of the safest and most efficient designs, many areas of GE optimization allow fundamental and active optimization. In addition to in silico or experimental identification and dismissal of basic gRNA designs, other options to avoid off-target GE include extended or truncated gRNAs, as well as the incorporation of modified nucleotides [47]. However, a powerful additional means of ensuring on-target fidelity, other than by safety and efficacy assessments, is through swapping or modifying the protein component to use alternative PAM sequences or reducing binding stability or editor activation for imperfect matches [66].

### 3.5. Engineering Advanced Editing Systems

Naturally occurring bacterial Cas enzymes, such as SaCas9, Cas12a, Cas3, or CoCas9, have inherently different binding and cleavage properties, including alternative PAM requirements. Discovery of alternative Cas nucleases such as the above-mentioned [67,68,69] and engineering of Cas nucleases with alternative PAM sequences has significantly expanded the target range [70]. Beyond PAM properties, using specially modified versions of Cas9 can make GE safer and more precise [66]. For instance, high-fidelity Cas9 enzymes are designed to reduce accidental cuts in the wrong places, while Cas9 nickases carry mutations in one of the Cas9 nickase domains, resulting in single-strand cuts instead of double-strand breaks, further minimizing unintended changes. High-fidelity Cas9 variants have been engineered using multiple complementary principles (see [71] and references therein), including the removal of unspecific DNA contacts (e.g., for eSpCas9) [72] or of a mismatch-stabilizing protein loop (e.g., for SuperFi-Cas) [73,74]. Meanwhile, deactivation of Cas9 double-nickase activity has not only lowered DSB-mediated cytotoxicity and allowed increased on-target specificity for double nickase approaches [75], but was also the basis for enhanced efficiency of more advanced DNA modifications. Indeed, the employment of nickase-based editors with additional enzyme properties for advanced precision editing is currently emerging as a major trend for the development of safer GE-based treatments using base editors or prime editors. Overall, editor choice and advanced designs help scientists achieve more accurate edits with less risk to the patient [76,77].

Efficient transient **CRISPR/Cas delivery** into cells is essential for GE to succeed. Regardless of the Cas variant employed, the choice of delivery system is a critical determinant of both efficiency and safety, as already highlighted above. Prolonged intracellular Cas9 activity increases the likelihood of unintended DNA modifications. Therefore, rapid degradation of Cas9 after performing the desired edit is key to reducing off-target effects and improving overall safety. To achieve this, scientists have designed editors that can be switched on and off [78], and generally favor transient delivery methods, where GE tools are present only temporarily. Both viral and non-viral transient approaches can be used, enabling efficient editing while minimizing unwanted effects. In contrast, integrating vectors, which insert genetic material permanently into the genome, are generally discouraged due to their increased risk of harmful mutations. Thus, by carefully selecting delivery strategies and controlling the duration of CRISPR activity, researchers can maximize desired edits while keeping GE precise and safe.

## 4. Regulatory Issues

Therapeutic application of GE needs to be regulated for safety and efficacy. Such regulation needs to be aware of the status quo and of technical developments for their clinical implications, in consideration of ethical issues, potential risks, assessment methods, and stringency of safety requirements.

### 4.1. Best Practices and Regulatory Guidelines for Responsible GE

GE-based therapy raises ethical concerns at the global, societal and individual level, and not every concern can be addressed by national or regional regulation. These concerns are as diverse as the fields of application and the patients and communities affected [79,80]. They include the risk of societal tendencies toward eugenics [81], of germline transmission and its irreversible long-term effects for humanity generations [82,83], and of inequity in design through bias in the underlying genetic and clinical studies [84,85] in terms of inequity in access through regional and national differences in regulatory approval and through bias in wealth and infrastructure distribution for what are expensive and complex technologies [86,87]. At the individual level, concerns include informed consent [88,89], risk–benefit assessment, and absolute safety considerations [90]. In line with the Hippocratic principle, safety of the individual, of communities, and the general population is paramount, so clinical application generally excludes germline editing and safety is the first criterion of clinical assessment [82,83]. However, assessing any of the above aspects for clinical application of GE tools requires the development of a strong regulatory framework. For instance, access to advanced therapies should be equitable to avoid discrimination based on socioeconomic status, and patient selection should be based on clear clinical eligibility criteria, such as disease severity, age, and health status [91]. Furthermore, GE should be strictly limited to therapeutic purposes, avoiding non-medical or germline modifications. To assess safety of GE applications, long-term monitoring is necessary to ensure safety of the patient. One more crucial aspect is to provide accurate information to the patient about procedure, potential risks and limitations of the GE approaches [92]. Hence, active and open involvement of relevant stakeholders—patients, ethicists, policy makers, and other stakeholders—is of critical importance for the proper integration of GE technologies with norms and values within a society. Likewise, appropriate prioritization of access to GE therapy must be performed as a means of minimizing health inequalities in circumstances of limited resources where cutting-edge medical technologies remain inaccessible [93], and creating and complying with regulatory frameworks is essential in fostering confidence and accountability in every GE application. Regulatory bodies such as the FDA and the EMA play a critical role in developing policies for clinical trials, guiding risk management strategies and approving new therapies [94,95]. Therefore, an understanding of principal differences and commonalities between FDA and EMA is instrumental in the establishment of advanced therapies with potentially global reach (see Table 2).

#### 4.1.1. FDA Regulatory Considerations

It is worth noting that FDA guidance defines human GE as the precise addition, deletion, modification, or replacement of DNA sequences at designated locations in human somatic cells, performed either ex vivo or in vivo, using technologies that may be nuclease-dependent or nuclease-independent [94]. Products that utilize these approaches are referred to as human GE products. The FDA assesses each such product using a science-based evaluation of its benefits and risks, considering the intended indication, patient population, anticipated magnitude and duration of therapeutic effect, and available treatment alternatives [94]. Major risks identified include unintended changes at non-target sites (off-target effects), unexpected outcomes at the target site, and unknown long-term effects of genome modifications. The FDA guidance also addresses critical aspects of GE products, including the methods used for GE, type and extent of genomic modifications, components of the editing process, and the delivery methods employed to introduce these components into target cells [94]. Each of these factors influences safety, efficacy, and regulatory evaluation of human GE products. This is essential for the FDA, which emphasizes the need for detailed characterization to inform risk–benefit assessments and clinical study design. Furthermore, the FDA provides Chemistry, Manufacturing, and Controls (CMC) recommendations, which build on general CMC guidance for gene therapy Investigational New Drugs (INDs). These recommendations include specific considerations for the design, manufacture, and testing of GE components as well as the final drug product for both in vivo and ex vivo applications, ensuring quality, reproducibility, and regulatory compliance [94]. The FDA recommends that clinical development programs address both the inherent risks of gene therapy products and the additional risks specific to GE, such as off-target effects and unintended on-target consequences [94]. Therefore, clinical trials should define patient population clearly and use a safe and efficient administration strategy (including dosing, schedule, and treatment plan), along with robust safety monitoring and appropriate efficacy endpoints. Long-term follow-up is advised to assess ongoing safety. In special considerations for research involving children, pediatric studies with more than minimal risk include an Institutional Review Board to ensure that potential risks are justified by anticipated direct clinical benefits, supported by adult or animal data. If children are included, older children or adolescents should generally be enrolled before infants, according to the specific context of the disease [94].

#### 4.1.2. EMA Regulatory Considerations

In the European Union (EU), the EMA is the central regulatory authority for advanced therapies. These include gene therapies and GE, which in the EU are referred to as advanced therapy medicinal products (ATMPs) as a summary term. EMA’s Committee for Advanced Therapies (CAT) reviews scientific evidence and provides recommendations for the approval of ATMPs, including GE therapies [95]. As a key misalignment, EMA, unlike the FDA as its United States counterpart, applies regulation for genetically modified organisms to GE-derived products, so that in the EU, approval of advanced therapies is subject to environmental risk assessment [97]. Like the FDA, the EMA ensures that GE interventions meet necessary regulatory standards for safety and efficacy and provides guidelines for clinical trials and marketing authorizations [94,95]. Furthermore, the EMA also emphasizes the importance of transparent and rigorous clinical trial design, as well as the importance of assessing potential risks and long-term monitoring of patients [95,96]. In this context, the EMA recognizes that GE is a rapidly evolving field, by following a risk-based approach and giving regulatory guidance for ATMPs that balances flexibility with strict manufacturing and control standards [95]. Product comparability must be carefully assessed, and, in some cases, additional non-clinical or clinical data may be required beyond quality studies. In this context, the personalized nature of certain GE therapies poses challenges for batch testing and release, particularly for small batches, prompting ongoing efforts toward international harmonization [95]. Moreover, process development and product characterization should explicitly consider both on-target and off-target genomic modifications to ensure safety, efficacy and consistent product quality. Regarding safety and toxicity, the EMA emphasizes the importance of comprehensively identifying both on-target and off-target effects following GE [95]. For instance, off-target effects are influenced by the quality of the editing tool, delivery system, DNA target, cell type and differentiation stage, chromatin structure, nuclease exposure duration, and route of administration (in vivo or ex vivo) [95]. Editing errors can include point mutations, insertions, deletions, and chromosomal rearrangements, while even on-target editing may cause small nucleotide errors, insertion of additional DNA sequences, or “genomic scarring,” particularly via spontaneously occurring repair by error-prone repair mechanisms [95,96]. Sensitive, unbiased methods—such as GUIDE-seq and CIRCLE-seq—as well as in silico models are recommended to detect and characterize these effects [95,96]. The EMA advises that multiple complementary methods be used to map on- and off-target effects, reporting sensitivity, specificity, stability, and clinical significance, with the required reporting details scaled according to the risk profile of the product [95].

The EMA emphasizes improving knowledge and expertise through closer interactions with stakeholders, including basic scientists, patients, healthcare professionals, and other sectors such as agriculture, to identify expertise gaps and possible ethical concerns. They propose building a European network of regulatory experts (EMRN) and developing a dedicated EU curriculum for GE. Regulatory framework improvements include adopting an integrated approach considering broader physiological and pharmacological effects, facilitating clinical trials—particularly in vivo applications—harmonizing definitions and classifications of GE products internationally, and clarifying the use of external controls and orphan status [95]. Collaboration with stakeholders is also encouraged, including early dialogue with HTA bodies, academia, patients, and industry. EMA with its regulatory framework further incentivizes data sharing according to the European Health Data Space principles, and expands international collaboration with regulators, including the FDA, to support global alignment and regulatory harmonization [95].

#### 4.1.3. EMA and FDA with a Broad Regulatory Consensus for GE

Adhering to the EMA and FDA regulatory frameworks and expectations is crucial for conducting ethical and safe clinical trials and for a streamlined road to approval of suitable therapies. Following this, Box 2 summarizes key steps aligned with FDA and EMA expectations for responsible clinical GE.

Both EMA and FDA guidelines strongly support patient welfare and safety [94,95]. The accurate application of these guidelines improves the safety profile of GE clinical trials, minimizing potential side effects. Specifically, these frameworks require comprehensive preclinical studies to evaluate the accuracy of the method and evaluation of off-target effects before progressing to human clinical trials. Moreover, EMA and FDA regulations strongly support continuous and long-term patient follow-up. This allows the evaluation of persistence and durability of therapeutic effects as well as the identification of late-onset side effects. The utilization of widely accepted, validated methodologies significantly increases quality of the procedure, resulting in high-quality data collection and analysis that ensures reliability and accuracy of trial outcomes. Additionally, establishment of ethical principles protects vulnerable populations and ensures informed consent. Adherence to regulatory recommendations enables the development of a common framework across countries that aligns with international and local requirements, avoiding trial delays. Overall, the adherence to international regulatory frameworks ensures development of a common approach to evaluating safety and efficacy of gene therapies, fostering patient safety [94,95].

Box 2 **FDA/EMA-aligned key steps for clinical GE.**

**Define intended clinical use and patient population.** Clearly specify the disease target, editing approach (ex vivo or in vivo), and expected clinical benefit, and explain how the anticipated benefit justifies the potential risks before initiating regulatory submissions.**Characterize editing tools.** Provide data on nuclease specificity, editing efficiency, *off-target* activity, and unintended *on-target* changes, together with delivery system safety, using appropriate in vitro, in vivo, and in silico assays.**Select safe and compliant delivery methods.** Use FDA/EMA-accepted delivery approaches (viral or non-viral) and dosing regimens supported by preclinical data, and justify route, schedule, and dose escalation in line with regulatory expectations for ATMPs.**Develop a strong Chemistry, Manufacturing, and Controls (CMC) package.** Document potency, purity, reproducibility, batch consistency, and scalability under Good Manufacturing Practice (GMP) conditions.**Design ethically sound clinical trials.** Clearly define eligibility criteria, endpoints, safety monitoring, and stopping rules, and introduce pediatric participants only when supported by prior data and ethical review.

6.**Implement rigorous monitoring.** Track *on-target* and *off-target* effects, immune responses, and long-term safety, with a follow-up duration appropriate to the risk profile and route of administration of the GE product.7.**Ensure patient equity and access.** Avoid unjustified socioeconomic or demographic biases in enrolment and prioritize fair and transparent criteria for access to experimental therapies.8.**Maintain transparent communication.** Report adverse events, protocol changes, and key safety and efficacy findings in a timely and complete manner to regulators, ethics committees, and, where appropriate, the scientific community.9.**Engage stakeholders early.** Involve regulators, patients, ethicists, and clinicians in protocol design for alignment on acceptable risk levels.10.**Promote regulatory harmonization.** Coordinate documentation, terminology, and data standards to be compatible with both FDA and EMA frameworks, facilitating multinational development and review of GE trials.

A notable example illustrating the differences in regulatory pathways despite broad consensus is *Valoctocogene roxaparvovec* (Roctavian), a gene therapy for severe hemophilia A. In the European Union, the EMA granted a conditional marketing authorization in 2022 [98], allowing earlier patient access while requiring continued data collection on safety and efficacy. In contrast, the U.S. FDA granted full approval in 2023 [99] only after the company provided comprehensive long-term data demonstrating durability and safety of the treatment [100]. This divergence highlights the EMA’s willingness to accept some uncertainty through conditional approval, whereas the FDA emphasizes more definitive long-term safety, preferring full endorsement only after additional data rather than partial endorsement. This case, alongside documented differences in trial data submissions [101] and unmet medical need prioritization programs [102], highlights distinct regulatory philosophies requiring strategic planning for global development. Understanding these differences is critical for developers aiming for global regulatory compliance and underscores the need for international harmonization efforts.

### 4.2. Challenges, Risks and Potential Solutions

As stated before, GE has the potential to revolutionize treatment of genetic diseases [103]. However, besides persistent inequity in access and affordability of advanced therapies [104], the path towards widespread clinical application of CRISPR-based technologies also critically depends on improvements in safety and efficacy and on the approval of corresponding treatments by regulatory authorities [105]. See Table 3 for exemplary GE products in development pipelines and in the context of corresponding FDA and EMA classification and approvals. Product approval of clinical interventions involving GE is based on the safety of the editing process itself, but also on efficient and specific delivery of editing tools into target cells [14], through viral and non-viral methods. Although viral vectors remain widely used due to high delivery efficiency, they raise safety concerns related to immunogenicity and insertional mutagenesis [14,41]. On the other hand, non-viral delivery systems, such as lipid nanoparticles, are attractive alternative delivery methods for reducing safety concerns related to off-target effects and cytotoxicity compared to viral vectors [106]. However, lower delivery efficiency to non-hepatic tissues remains a major challenge for the clinical application of nanoparticle-mediated GE systems [46]. To overcome these limitations, research should focus on developing more efficient, tissue-specific delivery systems that minimize cell immune responses and maximize precision. This includes, for example, the design of nanoparticles with higher target specificity or the development of next-generation viral vectors with reduced immunogenicity. Furthermore, exploration of physical, non-viral delivery methods, such as electroporation or microinjection, may provide viable alternatives in some contexts [107].

Manufacturing scalability represents another major challenge for clinical GE applications. The transition from small-scale laboratory experiments to large-scale clinical applications often presents difficulties in maintaining editing efficiency and minimizing off-target effects [128], so the development of scalable and standardized protocols for large-scale cell culture and methods for in vivo editing is crucial. Collaborative efforts among research institutions, biotechnology companies, and regulatory agencies are needed to establish best practices to ensure that GE techniques can be reproduced at scale while maintaining patient safety and to find efficient and cost-effective methods of production. Moreover, automation of the GE process and improved precision editing techniques at the tissue or organ level will help to overcome this challenge.

Although GE can produce immediate therapeutic benefits, it is also essential to understand and attend to long-term consequences of gene manipulation [129]. In this sense, long-term changes in gene expression or cell behavior may lead to unpredicted consequences [130]. Therefore, rigorous preclinical testing needs to be conducted using advanced high-resolution techniques to detect or anticipate genotoxic events in ex vivo and in vivo models, followed by broad, multi-ethnic human clinical trials that include long-term follow-up.

## 5. Methodology

This review is based on a comprehensive review of scientific literature and regulatory guidance available online, with a particular focus on identifying, evaluating, and synthesizing best practices for the responsible implementation of GE technologies in clinical contexts. A broad spectrum of peer-reviewed articles, reviews, clinical reports, and case studies was thoroughly examined to gather detailed information on clinical applications, safety considerations, regulatory frameworks, and practical recommendations. Searches were conducted across major scientific databases, including PubMed, Web of Science, and Scopus, using keywords such as “genome editing”, “CRISPR”, “clinical applications”, “safety guidelines”, “ethical considerations”, and “best practices”. Inclusion criteria emphasized recent, high-quality publications published in English over the past decade, ensuring that the review reflects the most current and scientifically robust evidence available.

In addition to scientific literature, publicly available official regulatory documents and guidance from the FDA and the EMA were carefully reviewed to incorporate current legal requirements, safety standards, and recommended procedures relevant to clinical GE. These documents provided essential context for the development of practical recommendations, helping to align scientific findings with regulatory expectations and internationally recognized standards.

Overall, this approach ensured that the information presented not only reflects scientifically verified evidence but also provides guidance for researchers and clinicians, promoting responsible, safe, and ethically informed use of GE technologies.

## 6. Conclusions

Despite progress, key gaps remain in GE translation, from conception to marketing approval. This starts with a greater need to focus on minimizing off-target effects and ensuring consistent delivery methods across diverse cell types and diseases, rather than on peak efficiency figures. On the researcher side, first contact with a regulatory agency is often delayed, resulting in the need to perform complementary analyses to conclude preclinical studies in line with regulations. On the side of regulatory agencies, there is a constant need to stay ahead of developments in a fast-moving field and to provide clear guidance for standardized long-term monitoring protocols to detect rare or delayed adverse events. Altogether, future efforts must prioritize the development of higher-precision editing tools and of robust and scalable delivery technologies, but also of internationally harmonized yet nimble regulatory frameworks. Finally, advancing equitable patient access—through diversity-aware design and practicable reimbursement models—and enhancing informed consent processes—especially for vulnerable populations, are essential to maintain public support and trust.

The success of GE in medicine thus relies heavily on safety, effectiveness, and, in its long-term sustainability, on ethical application. To achieve these standards, scientists must consider diversity, carefully optimize delivery, minimize unintended changes, and establish thorough monitoring plans both before clinical trials and over the long term to manage any potential risks. Diversity must be considered and in parallel, regulatory agencies, such as the FDA in U.S. and the EMA in Europe, should provide essential guidance. Despite scope for improvement in a still emerging field, evidence from case studies demonstrates that adhering to best practices and common principles across agencies enables GE to be integrated safely into clinical routine.

## Figures and Tables

**Table 1:** Overview of representative CRISPR design and off-target prediction and detection tools.

Tool	Approach Type *	Description/Advantages
COSMID	In silico (computational)	Scans the genome for potential off-target sites using the guide sequence, including small differences, insertions, or deletions. It also suggests DNA fragments for precise analysis in the lab [49].
CRISPOR	In silico	Helps design efficient CRISPR guides, predicts *off-target* sites, and scores guide efficiency across multiple genomes, reducing experimental errors [55].
Cas-OFFinder	In silico	Identifies potential CRISPR *off-target* sites without limitations on mismatches and considers PAM sequence variations, helping minimize *off-target* effects by design [56].
Breaking-Cas	In silico	Helps design efficient CRISPR guides, predicts and shows off-target sites in genomic locations, and allows any ENSEMBL build or current and future Cas parameters [57].
CRISPRroots	In silico (machine learning-based)	Combines CRISPR/Cas9 edits with RNA-seq data to detect *on-target* and potential *off-target* effects, using gene expression changes, guide RNA binding, and sequence variants [54].
GUIDE-seq	Experimental (cell-based)	Detects CRISPR/Cas9 *off-target* sites in living cells by incorporating a detectable sequence tag at double-strand breaks, allowing sensitive mapping and quantification [51].
CIRCLE-seq	Experimental (in vitro)	Uses circularized DNA and sequencing to detect genome-wide *off-target* effects rapidly and reproducibly, without requiring living cells or reference genomes [52].

* In silico methods (COSMID, CRISPOR, Cas-OFFinder, Breaking-Cas, CRISPRroots) use computational algorithms to predict off-target sites based on the guide sequence, sequence mismatches, and, in some cases, gene expression data [54,55,56]. These tools help design efficient and specific CRISPR guides while minimizing experimental work. Experimental methods (GUIDE-seq, CIRCLE-seq) directly detect off-target cleavage events in living cells or in vitro, providing high-sensitivity, genome-wide validation [51,52]. Together, these complementary strategies allow researchers to optimize CRISPR on-target specificity. For a more detailed and comprehensive review of tools for GE specificity optimization, see Kalter et al. [4].

**Table 2:** Comparison of FDA and EMA regulatory guidance on human GE across key scientific and clinical aspects [94,95,96].

Aspect	FDA	EMA
Definition	Human GE is the targeted addition, deletion, modification, or replacement of DNA sequences at defined sites in human somatic cells, performed either ex vivo or in vivo, through technologies that may rely on nucleases or operate independently of them.	GE is part of gene therapy products; regulatory guidance follows a risk-based approach addressing DNA modifications in human somatic cells ex vivo or in vivo.
Delivery/Methods	Emphasizes delivery strategies—viral or non-viral—the type and extent of genomic modification, and the specific editing components, noting that the chosen method directly influences both safety and efficacy.	Considers delivery and editing components as part of process development; personalized therapies may affect batch testing and release strategies.
*Off-target/On-target* Risks	Highlights *off-target* and *on-target* editing errors; recommends multiple complementary detection methods (in vivo, in vitro, in silico) with reporting of sensitivity, specificity, and clinical relevance.	Stresses identification of *on-* and *off-target* effects; errors include point mutations, insertions, deletions, translocations; recommends multiple unbiased methods (e.g., GUIDE-seq, CIRCLE-seq) and scaling risk mapping according to product profile.
CMC/Quality	Provides guidance on Chemistry, Manufacturing, and Controls (CMC), including GE component design, manufacture, testing, and drug product testing for in vivo and ex vivo products.	CMC follows a risk-based approach; comparability requires careful assessment, may need additional non-clinical/clinical data; batch testing for personalized products can be burdensome; *on-/off-target* modifications included in characterization.
Clinical Considerations	Recommends assessing product and GE-specific risks, defining patient population, safe administration, safety/efficacy endpoints, and long-term follow-up; includes special considerations for children.	Similar considerations; emphasize risk-based clinical trial design, early adult cohorts before pediatric enrollment, and long-term monitoring; *off-/on-target* effects integrated into safety assessment.

Concise side-by-side overview of the FDA and the EMA regulatory perspectives on human GE, meant to summarize key points from the main text, as an accessible reference for researchers, clinicians, and developers seeking to align GE therapies with current international regulatory expectations. For details, see the main text.

**Table 3:** Exemplary GE product pipelines for rare diseases and related classification and approvals.

Disorder ^1^	Target Gene	Editing/Delivery Approach ^2^	Therapeutic Goal	FDA Status ^3^	EMA Status ^4^	Product
AATD	*SERPINA1*	In vivo Adenine BE (LNP)	Correct the Pi*Z variant to restore functional AAT	OD (2025-05)	no OD; no MA	Beam BEAM-302 [108]
ATTR A	*TTR*	In vivo CRISPR/Cas9 knockout (LNP to liver)	Reduce circulating TTR to treat ATTR	OD (2021-10)	OD (2021-03)	Intellia NTLA-2001 [109]
B-cell AID (αCD19)	*TRAC*, *B2M*, *CIITA*, (+CD47)	Ex vivo CRISPR hypoimmune	Kill pathogenic B cells	FT	no OD	Sana SC291 [110,111,112]
B-cell M (αCD22)	*TRAC*, *B2M*, *CIITA*, (+CD47)	Ex vivo CRISPR hypoimmune	Kill CD22^+^ malignant B cells	IND	no OD	Sana SC262 [110,111,112]
HAE	*KLKB1*	In vivo CRISPR/Cas9 knockout (LNP to liver)	Lower Kallikrein to prevent HAE attacks	OD (2022-09); RMAT	OD designated	Intellia NTLA-2002 [113,114]
LCA10	*CEP290*	In vivo AAV-delivered CRISPR/Cas9 (dual gRNA)	Restore photoreceptor function	IND (2018-04); RPD (2024-01)	OD (2018-01)	Editas EDIT-101 [115]
CGD	*NCF1*	Ex vivo aHSPC EP for PE as RNA	Correct *NCF1* to restore NADPH oxidase	OD + RPD (2025-05); IND (2024-05)	no OD	Prime Medicine PM359 [116,117]
SCD	*HBG1*/*HBG2* promoters	Ex vivo aHSPC EP for ABE	Reactivate HbF	OD (May/Jun 2025); RMAT	no OD	Beam BEAM-101 [118]
SCD	*HBG1*/*HBG2* promoters	Ex vivo aHSPC EP for AsCas12a	Reactivate HbF	OD (2023-04); RMAT (2023-10)	no OD	Editas reni-cel/EDIT-301 [119,120,121]
SCD	*BCL11A*	Ex vivo aHSPC EP for CRISPR/Cas9	Reactivate HbF	MA (2023-08)	MA (2024-02)	Vertex/CRISPRTX CASGEVY/exa-cel [122]
TDBT	*HBG1*/*HBG2* promoters	Ex vivo aHSPC EP for AsCas12a	Reactivate HbF	OD (2023-01); RPD (2020-08)	no OD	Editas reni-cel/EDIT-301 [119,120,121,123,124]
TDBT	*BCL11A*	Ex vivo aHSPC EP for CRISPR/Cas9	Reactivate HbF	MA (2024-01)	MA (2024-02)	Vertex/CRISPRTX CASGEVY/exa-cel [122,125]
X-CGD	*CYBB* gene insertion	In vivo HSPC via VLP/HDAd to insert CYBB	Restore NADPH oxidase in neutrophils	RPD (2025-02); IND (2025-05)	no OD	Ensoma EN-374 [126,127]

This table presents exemplary GE product pipelines designed to address rare diseases, detailing their molecular targets, editing or delivery strategies, therapeutic objectives, and corresponding regulatory designations and approvals. Furthermore, it showcases representative investigational therapies that leverage a broad spectrum of cutting-edge editing and delivery platforms across U.S. FDA and EU EMA designations. Collectively, these emerging programs reflect the clinical maturation of both in vivo and ex vivo gene editing modalities, signaling a transformative shift in the treatment paradigm for monogenic and hematologic disorders—and highlighting GE as one of the most promising frontiers in modern rare disease therapeutics. ^1^ AATD: Alpha-1 antitrypsin deficiency; ATTR A: ATTR amyloidosis; B-cell AID: B-cell autoimmune disease; αCD19: CD19-targeting allogeneic CAR-T cell design; B-cell M: B-cell malignancies; αCD22: CD22-targeting allogeneic CAR-T cell design; HAE: Hereditary angioedema; LCA10: CEP290-related Leber congenital amaurosis 10; CGD: p47phox-related chronic granulomatous disease; SCD: sickle-cell disease; TDBT: transfusion-dependent β-thalassemia; X-CGD: X-linked (gp91phox) chronic granulomatous disease. ^2^ AAV: adeno-associated virus; BE: base editor; LNP: lipid nanoparticle; PE: prime editor; aHSPC: autologous hematopoietic stem and progenitor cell; EP: electroporation; ABE: adenine base editor; VLP: virus-like particle; HDAd: helper-dependent adenoviral vector. ^3^ All listed treatments are classified by the FDA as cellular or gene therapy products (CGTP) across the following designations and approval stages. FT: Fast Track; IND: Investigational New Drug; MA: marketing approval; OD: orphan drug; RMAT: regenerative medicine advanced therapy; RPD: Rare Pediatric Disease. ^4^ All listed treatments are classified by EMA as the advanced therapy medicinal product (ATMP) subclass gene therapy medicinal product (GTMP).

## Data Availability

No new data were created or analyzed in this study. Data sharing is not applicable to this article.

## Abbreviations

The following abbreviations are used in this manuscript:

GE	Genome Editing
ZFN	Zinc-Finger Nuclease
TALEN	Transcription Activator-Like Effector Nucleases
CRISPR	Clustered Regularly Interspaced Short Palindromic Repeats
Cas	CRISPR-Associated Proteins
FDA	Food and Drug Administration of the United States
U.S.	United States
EMA	European Medicines Agency
DNA	Deoxyribonucleic Acid
RNA	Ribonucleic Acid
RNP	Ribonucleoprotein
CPS1	Carbamoyl-Phosphate Synthetase 1
DSB	Double-Strand Breaks
NK	Natural Killer
KRAS	Kirsten Rat Sarcoma Viral Oncogene Homolog
HSPC	Hematopoietic Stem and Progenitor Cell
IDLV	Integrase-Defective Lentiviral Vector
AAV	Adeno-Associated Virus Vector
VLP	Virus-Like Particle
LNP	Lipid Nanoparticle
EV	Extracellular Vesicle
gRNA	guide RNA
PAM	Protospacer Adjacent Motif
CMC	Chemistry, Manufacturing and Controls
IND	Investigational New Drug
CAT	Committee for Advanced Therapies
EU	European Union
ATMP	Advanced Therapy Medicinal Product
RMAT	Regenerative Medicine Advanced Therapy
RPD	Rare Pediatric Disease
OD	Orphan Drug
HTA	Health Technology Assessment
EMRN	European Network of Regulatory Experts
GMP	Good Manufacturing Practice
